# Toxic effect of acyclovir on testicular tissue in rats

**Published:** 2013-02

**Authors:** Elham Movahed, Vahid Nejati, Rajabali Sadrkhanlou, Abbas Ahmadi

**Affiliations:** 1 *Departmant of Biology, Faculty of Basic Sciences, Urmia University, Urmia, Iran.*; 2 *Laboratory of Embryology, Department of Basic Sciences, Faculty of Veterinary Medicine, Urmia University, Urmia, Iran.*

**Keywords:** *Acyclovir*, *Testis*, *Testosterone*, *Mast cells*, *Rat*

## Abstract

**Background: **Acyclovir (ACV), a synthetic purine nucleoside analogue, is known to be toxic to gonads.

**Objective:** The current study evaluated cytotoxicity of ACV on histopathological changes in testis tissue and serum testosterone and lipid peroxidation concentrations of male rats.

**Materials and Methods:** Animals were divided into five groups. One group served as control and one group served as control sham. In the drug treated groups ACV administered for 15 days. 18 days after the last injection, animals were sacrificed. Histopathological and histomorphometrical analysis of the testis was carried out. Serum levels of testosterone and Lipid Peroxidation and potential fertility of animals was evaluated.

**Results:** Male rats exposed to ACV had significant reduction in serum testosterone concentrations at 16 and 48mg/kg dose-levels (p<0.01). ACV induced histopathological changes in the testis and also increase the mean number of mast cells in peritubular or interstitial tissue in the testis at at 16 and 48mg/kg dose-levels (p<0.01). In addition ACV caused increase of serum level of Lipid Peroxidation at 48mg/kg dose-level (p<0.05). As well ACV decreased potential fertility in male rats.

**Conclusion:** The present results highly support the idea that ACV has adverse effect on the reproductive system in male rat.

## Introduction

For over two decades, the synthetic acyclic purine nucleoside acyclovir (ACV), considered as the first choice of treatment for herpes simplex virus types 1 and 2 (HSV-1 and HSV-2). In addition it has been reported to be very effective against the treatment of varicella zoster infection and it also protects immunosuppressed patients who were receiving transplants from cytomegalovirus (1, 2). ACV inhibits viral DNA replication effectively much more than cellular DNA replication indicating that ACV can mildly impair host cell (3-5). 

In a series of in vivo and in vitro studies, ACV was found to be clastogenic to somatic cells. It causes micronuclei formation in in vitro or in vivo mouse indicating that it is also capable to damage cellular DNA in the non-infected cells (6-8). ACV is reported to inhibit cell division in human fibroblast cell and increase in the chromosomal damage in the human lymphocyte (9, 10). It has previously been demonstrated that exposure of HeLa cells to ACV led to significant increase in the lactate dehydrogenase (LDH) (1). As well, it causes LDH releasing from the cells of the testis tissue and increase in the serum LDH concentration in patient receiving this drug (11, 12). All of these studies suggesting cytotoxicity of ACV. Moreover, ACV has reported to induce decreased interatesticular testosterone level and increase sperm abnormal parameters in mice (11).

It is well known that reproductive system is very sensitive to toxic chemicals because of the high multiplication rate of germ cells that result in high susceptibility of male gonad to toxic effect of chemotherapy (13, 14). On the other hand this is the only system in which transmissible genetic damage from one generation to another takes place (15). There is a few studies on the influence of ACV on reproductive performances in male of different species.

Therefore, the aim of the present investigation was to study the effect of various concentrations of ACV on serum testosterone and Lipid Peroxidation concentrations and testicular tissue of adult male rats treated with this drug. In this study unlike other investigations several factors was evaluated simultaneously including serum testosterone level because of its importance for spermatogenesis and fertility, serum Lipid Peroxidation level because of its role in toxicity and carcinogenisity of testis, number of mast cells in the testis tissue because of its relation to male infertility and other histological and histomorphometrical parameters in testis tissue (16-21).

## Materials and methods

**Drug and animal treatment**

ACV (purchased from MYLAN Company, France) was used at three dose levels, 4, 16 and 48 mg/kg based on previous studies (11). Drug was dissolved in distilled water before injection. Forty male Wistar rats (220±20 g) were obtained from animal house of Faculty of Science, Urmia University, and kept under specific conditions on a constant 12-hour light/dark cycle and at a controlled temperature of 22±2^o^C. Standard pellet food and tap water were given ad libitum. Animals were allowed to acclimatise for one week before experimental use. 

They were segregated into five groups of eight rats each Group 1 served as control, normal and apparently healthy rats that did not receive any type of treatment. Group 2 served as control sham and received distilled water (intraperitoneal (i.p.) injection) once a day for 15 consecutive days to evaluate any effect of i.p. injection on purposed parameters of present study. Groups 3, 4 and 5 (the drug treated groups) administered 4, 16 and 48 mg/kg/day ACV (i.p. injection) respectively once a day for 15 consecutive days. It should be noted that this study was an experimental study accordance with the guidance of ethical committee for research on laboratory animals of Urmia University.

18 days after the last injection, animals (4 animals from each group) were weighed, and were sacrificed by CO_2_ inhalation. The blood samples were collected from jugular vein. The blood was centrifuged and subsequently the serum was harvested and frozen (at -80^o^C). The left testes were removed and weighed.

**Potential fertility assay**

One week before the end of the treatment period, four males from each group was placed in an individual cage with two super-ovulated same strain females. The presence of vaginal plugs and as well observation of sperm in the smears in the following morning was an indication, that mating had occurred and this was designated day 1 of gestation. For each group the number of pregnant female rats and also the number of offspring recorded. 

**Histopathological examination**

The removed tissues from males were fixed in 10% neutural buffered formalin and processed for paraffin embedding. Five µm thick sections were stained with Haematoxylin and Eosin (H&E) for histopathology, some sections with Wigert’s Iron Haematoxylin for evaluation of Repopulation Index of spermatogonia and other sections were stained with Toluidine Blue for mast cell identification. 

**Histomorphometrical analysis**

For each testis, in 20 randomly selected tubular profiles that were round and nearly round, the diameters of tubules (STD), epithelial height (SE) and interstitial connective tissue between tubules (CT) were measured by light microscopy. For the estimation of spermatogenesis in testicular tissue, three different indices were used. Tubular differentiation index (TDI), repopulation index (RI) and spermiogenesis index (SPI). 

To determine the tubular differentiation index, the number of seminiferous tubules that have more than three layers of germinal cells derived from type A of spermatogonia was calculated. To find out the repopulation index, the ratio of active spermatogonia to inactive spermatogonia was calculated and to determine the spermiogenesis index, the ratio of the number of seminiferous tubules with spermatozoids to the empty tubules was calculated. Mast cells after staining appear purple in colour, and the mean number of mast cells (peritubular or interstitial) was estimated in 20 high-power fields (400X).

**Testosterone assay**

Serum testosterone concentrations were measured by using a testosterone Electrochemiluminescence Kit (Roche, Germany).

**Malondialdehyde assay**

Lipid peroxidation (LPO) in the serum was measured by the thiobarbituric acid-reacting substance (TBARS) and was expressed in terms of malondialdehyde (MDA) content (22). The method is based on the reaction of MDA with thiobarbituric acid (TBA) followed by the condensation of two molecules of TBA with one molecule of MDA and elimination of two molecule of water to obtain a TBA pigment (23).

**Statistical analysis**

The data are presented as the mean±SEM. Differences between groups were analyzed by One Way Analysis of Variance (ANOVA) followed by Tukey test using SPSS software, version 6.0 and level of significance was taken as p<0.05 or p<0.01. 

## Results

**Body weight and left testis weight**

There were no significant changes in the body weight and the relative left testis weight at any dose-level of ACV compared with those of control and control sham groups.

**Histology and histomorphometry of testis**

Histological and histomorphometrical examination of the testis by H&E technique revealed that ACV compared with those of control and control sham groups, markedly reduces STD at 16 and 48 mg/kg dose-levels (atrophy of tubules) and SE at all dose-levels in a dose dependent manner. As well, CT was significantly increased at 16 and 48mg/kg dose-levels in a dose dependent matter (Table I). 

H&E technique also showed significant decrease in TDI and SPI in the seminiferous tubules at all dose-levels when compared with the control and control sham groups in a dose dependent manner (Table II). 

**Figure 1:**
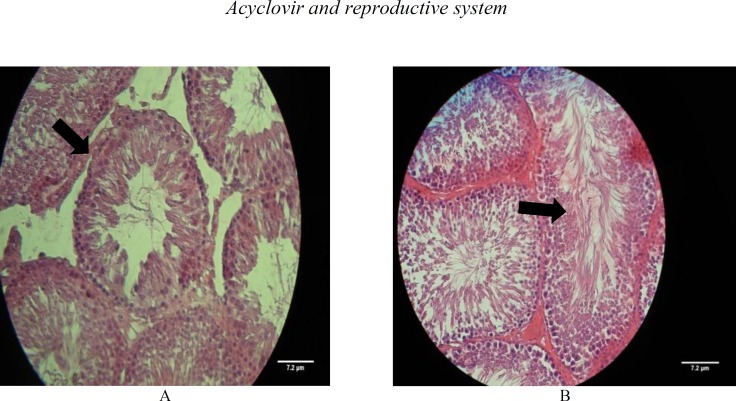
**a.** Cross section of testis from a treated rat with ACV (46mg/kg) shows some histopathological changes in the seminiferous tubules (H & E ×400). **b.** Cross section of testis from a rat of control group indicating normal cell association with no histological changes in germinal cell proportion (H & E ×400).

**Figure 2:**
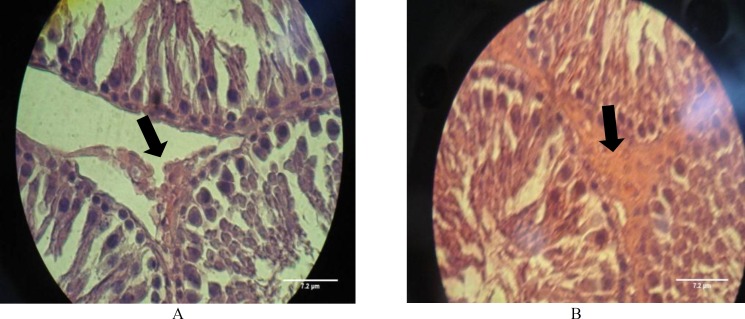
**a.** Cross section of testis from a treated rat with ACV (46mg/kg) shows Leydig cells atrophy in the interstitial tissue (H & E ×1000). **2b. **Cross section of testis from a rat of control group indicating normal Leydig cells association (H & E ×1000).

Some of these changes have been shown in Figure 1a. In addition, this technique demonstrated histopathological changes in the testis such as epithelial sloughing in some tubules and Leydig cells atrophy in the interstitial tissue in rats treated with 16 and 48 mg/kg dose-levels of ACV (Figure 2a), whereas there were no histopathological change in the control and control sham groups (Figure 1b, 2b). 

The Wigert’s Iron Haematoxylin technique showed ACV decreases RI at 48mg/kg dose-level of ACV. The mean number of mast cells in peritubular or interstitial tissue in the testis was increased at 16 and 48 mg/kg dose-levels compared to control and control sham groups as revealed by the Toluidine Blue technique (Table II).

**Potential fertility**

Female mated by male rats exposed to ACV showed significantly lower pregnancy rate. There were no significant changes in the mean number of the offspring at any dose-level of ACV as compared to those of control and control sham groups.

**Testosterone concentration**

Serum testosterone level was significantly increased at 16 and 48 mg/kg dose-levels of ACV compared with the control and control sham groups in a dose dependent manner (Table III).

**Malondialdehyde concentration**

The level of LPO in the serum in terms of MDA content was significantly increased at 48 mg/kg dose-level of ACV compared with the control and control sham groups (Table III).

**Table I:** Effects of i.p. administration of ACV on STD, SE and CT of seminiferous tubules in adult male rats

	**STD (µm)**	**SE (µm)**	**CT(µm)**
Control	233.374±8.050	43.723±1.113	8.775±0.488
Control sham	230.446±4.248	43.693±0.439	8.968±0.536
4 mg/kg ACV	222.375±5.920	32.695±0.378^a**^	11.463±2.145
16 mg/kg ACV	206.386±4.095^a*^	31.238±1.433^a**^	14.210±1.496^a*^
48 mg/kg ACV	197.613±4.572^a**b*^	27.053±1.032^ab**^	22.737±0.747^ab**^

Data are presented as mean±S.E.M with Tukey test from 4 animals per group.

*:p<0.05 **:p<0.01

STD: seminiferous tubules diameter.

SE: epithelial height.

CT: interstitial connective tissue between tubules.

^a^:significant compared with control and control sham.

^b^: significant compared with 4mg/kg ACV.

^c^: significant compared with 16mg/kg ACV^ .^

**Table II:** Effects of i.p. administration of ACV on RI, TDI, SPI and mean of mast cells in testis tissue in adult male rats

	**RI (%)**	**TDI (%)**	**SPI (%)**	**Mean number of** ** mast cells**
Control	84.837±1.587	94±0.912	92.25±1.108	0.832±0.166
Control sham	83.905±1.387	94±0.816	93.25±1.376	0.999±0.199
4 mg/kg ACV	81.461±1.066	58±2.449^a**^	63.50±3.304^a**^	1.333±0.272
16 mg/kg ACV	78.443±1.817	49±2.380^a**b*^	55.50±4.573^a**^	2.166±0.166^a**^
48 mg/kg ACV	77.464±1.380^ab*^	47±2.51^a**b *^	48.00±2.581^a**b*^	2.332±0.333^a**^

Data are presented as mean±S.E.M with Tukey test from 4 animals per group.

*:p<0.05 **:p<0.01

TDI: Tubular differentiation index.

RI: Repopulation Index.

SPI: Spermiogenesis Index.

^a^:significant compared with control and control sham.

^b^: significant compared with 4mg/kg ACV.

^c^: significant compared with 16mg/kg ACV^ .^

**Table III:** Effects of i.p. administration of ACV on serum testosterone and MDA level in adult male rats

	**Testostrone (ng/ml)**	**MDA (µmol/g)**
Control	3.365±0.257	0.098±0.018
Control sham	3.147±0.207	0.159±0.023
4mg/kg ACV	2.808±0.175	0.168±0.024
16mg/kg ACV	1.629±0.181^ab**^	0.32±0.107
48mg/kg ACV	0.945±0.138^ ab**^	0.344±0.035^a*^

Data are presented as mean±S.E.M with Tukey test from 4 animals per group.

*:p<0.05 **:p<0.01

MDA: Malondialdehyde

^a^:significant compared with control and control sham.

^b^: significant compared with 4mg/kg ACV.

^c^: significant compared with 16mg/kg ACV.

## Discussion

The present study was designed to demonstrate possible adverse effects of ACV on reproductive system and potential fertility in male rats. ACV was administrated at doses of 4, 16 and 48 mg/kg. These doses where chosen according to a preparative study at doses of 0, 4, 16, 32 and 48 mg/kg body weight of ACV, which investigated the effects of the various doses of ACV on male reproductive system in the male mice (11). Additionally one group was serving as control and one group was serving as control sham. 

Male rats exposed to ACV did not show any significant change in body weight and also in relative testis weight compared to control and control sham groups indicating that ACV does not affect the general health conditions in male rats. A sensitive and indispensable method for releaving disturbances in spermatogenesis is histopathological examination (24). 

Histological and histomorphometrical examination in this study showed that testes of rats in the control and control sham groups have normal histological picture with active spermatogenesis. However ACV significantly decreases STD and SE of seminiferous tubules due to cell loss from the epithelium and increases CT between seminiferous tubules and also causes Leydig cells atrophy and epithelial sloughing in some tubules. 

Moreover the results of this study demonstrated decrease in RI at the highest dose of ACV and decrease in the TDI and SPI at all doses. On the other hand hormonal analysis of this study showed decreased serum testosterone level in rats administrated with two higher doses of ACV. In an investigation Leydig cells have been reported to play a crucial role in testosterone synthesis (25). 

In another study use of a Leydig cell toxicant, 1, 2-dimethane sulfonate (EDS), to kill Leydig cells, resulted in decreased interatesticular testosterone level in rats (26). On the other hand, testosterone supports spermatogenesis, sperm maturation and sexual function, thus any disruption in testosterone biosynthesis can adversely affect male fertility (27). As well, it has been reported that removal of testosterone from the rat testis results in increased germ cell apoptosis (26). 

Also testosterone can affect Sertoli cells function and germinal cell degeneration and dislocation could take place due to damage in function of Sertoli cells and decreased testosterone level has been reported to enhance premature detachment of epithelial cells (28, 29). In addition, epithelial sloughing is an indicator of Sertoli cells damage (29). 

Atrophy of Leydig cells can be responsible for reduction in serum testosterone level. The changes in the seminiferous tubules, as observed during histopathological examination, may cause in result of hormonal effect and not consequence of a direct effect. As well increase in CT between seminiferous tubules is consequence of atrophy and decreased STD of tubules. Moreover spermatogonia are very sensitive to toxins that interfer with DNA replication due to several mitotic divisions that this cells have andergone (30). ACV inhibits DNA replication (12), thus it also can impair to spermatogonia cells as in this study releaved by decreased RI.

Mast cells, free cells type drive from haemotopoetic stem cells that usually found in connective tissues, are key effector cells in allergic reactions and IGE associated immune responses (31, 32). There are some evidences that in cases with idiopathic infertility, the number of mast cells were increased (18). As well in several studies it has been reported that increase in the number of mast cells in the testis tissue related to male infertility (18-21). In addition abnormal spermatogenesis was reported in association with increase in the number of mast cells in testis tissue (33). The present study showed that the mean number of mast cells in peritubular or interstitial tissue was significantly increased in the testis by administration of ACV at two higher doses meaning that this change could be related to decreased potential fertility in male rats exposed to this antiviral drug. 

It is well known that Reactive Oxygen Species (ROS) play a functional role as second messengers in many cell types (34) and it has been reported that LPO, one of the main manifestation of oxidative damage, plays an important role in toxicity and carcinogenisity of testis (17). In another study it has been found that increased LPO can change cellular membrane structure and then block cellular metabolism (35). As well oxidative stress prevents androgenesis by Leydig cells in testis tissue and also plasma membrane of the mammalian sperm is sensitive to ROS-related lipid peroxidation, in fact lipid peroxidation that is formed by ROS can damage cellular structure, motility, survival and metabolic functions of sperm (34, 36). The results of this study revealed that the highest dose of ACV lead to increase in the serum MDA level, that is an important indicator of LPO, in male rats (16). This can be responsible for some reproductive disorders observed in this investigation. 

## Conclusion

In conclusion present study suggests that ACV plays negative roles on reproductive system and function in male rats. ACV induces reproductive disorders in a dose dependent manner as revealed by decrease in the STD, SE, RI, TDI and SPI of seminiferous tubules and increase in the CT between seminiferous tubules, Increase of the mean number of mast cells in the testis tissue, reduction of serum testosterone level, increase in serum MDA level and finally reduction in the potential fertility of adult male rats.
